# Body condition-linked alterations in dog owners' visual prosody

**DOI:** 10.3389/fvets.2026.1962197

**Published:** 2026-10-28

**Authors:** Anna Gergely, Lőrinc András Filep, Tajda Kukovič, Anna Kis

**Affiliations:** 1 “Momentum” Psychobiology Research Group, Institute of Cognitive Neuroscience and Psychology, HUN-REN Research Center for Natural Sciences, Budapest, Hungary; 2 Department of Ethology, Eötvös Loránd University, Budapest, Hungary; 3 Doctoral School of Biology, Eötvös Loránd University, Budapest, Hungary; 4 Department of Psychology, Pázmány Péter Catholic University, Budapest, Hungary; 5 Faculty of Education, University of Ljubljana, Ljubljana, Slovenia

**Keywords:** dog-human interaction, FaceReader, facial expression, obesity, prosody

## Abstract

**Introduction:**

Visual prosody, the facial component accompanying speech, plays an important role in human social communication and is also a characteristic feature of dog-directed communication. Previous studies have shown that dog-directed visual prosody shares several features with infant-directed communication, although it also exhibits characteristics that differ between dogs and infants. Obesity has been associated with altered emotional processing and expression in humans, yet its potential effects on visual prosody have not been investigated. In the present study, we examined whether obesity is associated with variation in visual prosody during dog-directed communication.

**Methods:**

We analysed video recordings of 146 adult speakers interacting with their own dogs, infants, or adult human partners in semi-naturalistic settings, meaning that they were given instructions what to do, but these instructions were aimed to mimic real-life situations. Facial movements were quantified using automated facial coding software (Noldus FaceReader), and body shape was visually scored based on standard procedure. Linear mixed models were used to analyse Happy facial expression, facial Arousal, Valence, and activation of the cheek raiser muscle (AU6).

**Results:**

Body condition-related differences depended on both the communication partner and the facial variable examined. Lower facial arousal was detected in speakers with higher body condition scores across all communication contexts. In contrast, differences in positive facial expressions were specific to dog-directed communication: speakers with higher body condition scores displayed less intense Happy facial expressions and lower facial Valence when interacting with dogs, whereas no such differences were observed in infant- or adult-directed communication. Unexpectedly, higher body condition scores were associated with stronger cheek raiser (AU6) intensity during infant- and adult-directed communication, but not during dog-directed communication, which might be a species-specific compensation mechanism. In addition, replicating previous findings, female speakers displayed more intense positive facial expressions and facial arousal than males, although these gender differences were reduced in dog-directed interactions.

**Discussion:**

Our findings suggest that body condition is associated with variation in visual prosody, but these effects depend on the interlocutor and particularly pronounced during dog-directed communication. Different interpretations of these findings are presented in the discussion: altered mapping of muscle movement to FaceReader output, differences in FaceReader model quality, differences in habitual communicative style or expressivity, the amplifying or masking effect of communication differences, and a possible compensatory mechanism. The importance of these body condition-related alterations on the effectiveness of face-to-face communication, as well as social relationships need to be discussed.

## Introduction

Communication between humans and companion animals has many facets. Prosody is classically defined as a suprasegmental aspect of speech, including intonation, rhythm, fundamental frequency, loudness, and tempo (1). During natural communication these vocal properties co-occur with from gestures and facial movements accompanying utterances. This latter component is termed visual prosody. Visual prosody in dog-directed communication seems to convey emotional information, friendly intent, and helps to establish and maintain attention (2). It resembles the visual prosody of infant-directed speech in some ways, e.g., more exaggerated facial movements (2) or distinct facial expressions (3) compared to adult-adult communication, yet it also differs from it, e.g., in the Happy expression being less intense and decreased muscle activation in general (2, 4).

Studies of human children shed light on the importance of proper emotional communication, as the development of children who do not receive appropriate emotional signals from their primary caregivers tends to be impaired (5, 6). For example, children of depressed mothers may show delays in cognitive development, and behavioral effects such as more frequent and prolonged crying have also been reported, particularly during the first years of life (6). The relationship between pet dogs and their humans is comparable to that of an infant and its caregiver because they tend to form similar attachment relationships (7, 8), and companion dogs may even occupy a child-like role within the family (9). Furthermore, dogs are attuned to human communicative signals, as demonstrated in studies on gaze following (10) and the comprehension of pointing gestures (11, 12). In parallel with the extensive literature showing that parenting style and parental characteristics influence child development [e.g., (13)], dog training methods (14) and caretaker characteristics (15) also affect dog welfare and the quality of the dog-owner relationship. It is therefore reasonable to assume that visual prosody displayed by owners during dog-directed communication may influence both communication efficiency and the welfare of the dog-human dyad.

Dogs are known to extract extensive social information from human faces, including emotional content (16). Human studies also indicate that facial emotion recognition is sensitive to the visibility of facial features. For example, Barnett et al. (17) found that covering the lower half of the face impairs emotional processing, although the magnitude of this effect varies across emotions. Apart from artificial coverings, naturally occurring anatomical characteristics may also alter the visibility and movement of facial muscles involved in emotional communication. Obesity is an increasingly common condition in Western societies (18), and the accumulation of adipose tissue in facial regions could potentially modify the appearance or dynamics of facial expressions. Despite the growing prevalence of obesity, its possible effects on visual prosody and, consequently, on dog-human and human-human communication have not yet been investigated.

Beyond anatomical considerations, obesity appears to have a complex relationship with emotional processing and expression. Overweight and obese individuals often report greater difficulties in identifying, describing, and regulating emotions (19–21). Difficulties in emotional regulation may in turn influence emotional expression. In a recent study, negative attitudes toward emotional expression, particularly the belief that emotions should be controlled or suppressed, predicted higher body mass index (22). Similarly, expressive suppression has been linked to overeating and elevated BMI (23). Furthermore, Soussignan et al. (24) reported reduced facial emotional responses to desirable food stimuli in overweight and obese adults compared to normal-weight individuals. Although studies specifically investigating visual prosody in overweight or obese populations are currently lacking, these findings suggest that obesity may be associated with altered facial expressivity. Finally, there is also evidence that overweight facial appearance can influence the perception and interpretation of emotional expressions (25, 26), highlighting the multifaceted relationship between obesity and socio-emotional communication.

In the present study, we investigated whether overall body condition is associated with differences in visual prosody during infant- and adult-directed communication, as well as during dog-directed communication. The aim was to investigate possible effects of general body composition to visible, detectable facial movements. We analyzed video recordings of male and female speakers interacting with their pet dogs in naturalistic settings and compared these interactions with infant- and adult-directed communication. We hypothesized that speakers with higher body-condition scores would display lower intensity facial prosodic signals than speakers with lower body condition scores. This prediction was based on previous findings suggesting altered emotional processing, greater expressive suppression, and reduced facial responsiveness among overweight and obese individuals (22–24). We further expected that the relationship between body condition and visual prosody would vary across communication partners because facial prosody differs between dog-, infant-, and adult-directed communication (2, 4). Given the lack of previous studies addressing this question, however, we did not formulate specific directional predictions regarding the interaction between body shape and communication context.

## Methods

### Ethical statement

This research was approved by the Hungarian United Ethical Review Committee for Research in Psychology (EPKEB; No. 2022-85). All subjects volunteered to participate in the study and signed written informed consent.

### Subjects

Participants were *N* = 146 adult humans (51 males, 95 females; within the range of 18–59 years old) who had a 6–18 month old infant and an adult dog. Puppy dogs (below 1 year of age) were not included in the study as vocal prosody directed to puppies vs. adult dogs is known to differ (27). Two speakers did not provide meaningful data (i.e., the FaceReader did not detect their facial movements) therefore they were excluded and the final dataset contained measurements from 144 speakers. The video footage analyzed here were originally recorded for previous studies (2, 4, 28), and for an additional study that is not yet published (Koós-Hutás et al., in prep). Different smartphone cameras were used to record speakers' faces: a Samsung Galaxy A70, a OnePlus Nord N10 5G, and an Apple iPhone SE (2020, 2nd generation). The Samsung Galaxy A70 was equipped with a 32-MP (f/1.7) main camera and supported video recording at a maximum resolution of 3,840 × 2,160 pixels (4K/UHD) at 30 frames per second (fps). The OnePlus Nord N10 5G featured a 64-MP (f/1.79) main camera and supported a maximum video resolution of 3,840 × 2,160 pixels (4K) at 30 fps. The iPhone SE (2020) was equipped with a 12-MP (f/1.8) main camera and supported video recording at a maximum resolution of 3,840 × 2,160 pixels (4K) at 24, 25, 30, or 60 fps.

In most recordings [data from Gergely et al. (2), Koós-Hutás et al. (4), and Koós-Hutás et al., in prep; total *N* = 104], speakers were interacting with (1) their own 6–18 months old infant (infant-directed condition, ID), (2) their own adult family dog (dog-directed condition, DD), or (3) another adult human (their spouses or an opposite gender experimenter, adult-directed condition, AD). The order of the conditions were balanced across speakers. Data from Filep et al. (28), contain only female speakers in dog-directed condition (*N* = 42). In all recordings speakers were instructed to talk to their partners in a natural way (i.e., play a game with them, solve an easy task with them, teach them an object label or tell them a nursery rhyme). They were all facing the interlocutor, not the camera directly. It is important to note that situational differences are out of scope of the present study [for contextual details see Gergely et al. (2), Koós-Hutás et al. (4), and Filep et al. (28)]. The length of the recordings varied between minimum 20 s to maximum 5 min. Recordings took place in the subjects' home (2, 4) (Koós-Hutás et al., in prep), or in the laboratory at HUN-REN Research Center for Natural Sciences (28). The subjects were speaking in Hungarian, their native language.

### Analysis of facial movements

The Noldus Face Reader (version 9.0; Noldus Information Technology B.V., Wageningen, Netherlands) was employed to analyze facial movements. The program operates based on Ekman's Facial Action Coding System (29), i.e., registers individual muscle movements and assigns an intensity value to it. Its validity has been tested by Lewinski et al. (30) and Skiendziel et al. (31). We used the default settings of the program to analyze following variables:

- Happy facial expression: intensity (0–1) of Happy emotion that dominates during those social interactions we used in our dataset (2, 4, 28).
- Arousal: indicates the overall activation level of facial movements (0–1).
- Valence: implies positive or negative deviation from neutral (0) emotional state (−1–1).
- Action unit 06—Cheek raiser: intensity of cheek raiser muscle (0–1) that is involved in forming the Duchenne smile [e.g., (32, 33)].

### Body composition score

Body condition was assessed by body shape contour based on the available video recordings. Body condition rating was carried out by an experimenter (LF) blind to FaceReader outputs. An 8-point Likert-scale was used ranging from 1 = severe thinness to 8 = class III obesity (Table 1). This classification is in line with the guidelines of the Expert Panel on the Identification, Treatment of Overweight, Obesity in Adults (1998). As our sample was randomly collected, the distribution of speakers among obesity scores 1–8 are not counterbalanced. For the purpose of statistical analysis subjects were grouped into two body condition categories: (1) Normal weight (speakers with 1–4 obesity scores, *N* = 83) and (2) Overweight (speakers with 5–8 obesity scores, *N* = 63).

**Table 1:** Number of subjects per Body Condition Score categories.

Obesity score	Obesity category	Description	Corresponding BMI	No. of subjects
1	1 Normal weight	Severe thinness	< 16	*n* = 0
2		Moderate thinness	16–17	*n* = 0
3		Mild thinness	17–18.5	*n* = 9 (*n* = 6 male)
4		Normal	18.5–25	*n* = 74 (*n* = 26 male)
5	2 Overweight	Overweight	25–30	*n* = 28 (*n* = 8 male)
6		Obesity class I.	30–35	n = 24 (*n* = 8 male)
7		Obesity class II.	35–40	*n* = 9 (*n* = 3 male)
8		Obesity class III.	40 <	*n* = 2 (both female)

Interrater agreement on Body Condition scoring was assessed by independent coding of *N* = 13 recordings (by L.A.F. & T.K.) and resulted in an Intraclass Correlation of ICC = 0.861 (*p* = 0.001). Note that agreement between raters does not validate the scoring system itself further to as described in the cited guidelines.

### Statistical analysis

R studio (Posit Software, PBC, Boston, MA, USA; https://www.rstudio.com/) was used for the statistical analysis [R 4.2.3 (R Core Team, Vienna, Austria) using RStudio 2023.12.0 + 396]. Due to computational limitations associated with the full dataset (i.e., full dataset contained 964,610 rows (one observation refers to one frame which is 0.25 s) and 46 variables), analyses were performed on a simple random subsample (10% of observations, sampled without replacement) balanced for the obesity category and speaker identity (i.e., random sampling equally from each represented obesity score and each participant was represented). Simple random sampling provides each observation with an equal probability of selection and is an established approach for reducing large datasets while preserving unbiased population representation, provided that sample representativeness is verified [e.g., (34, 35)]. Our final dataset contained 96,461 rows and six variables (Happy, Neutral, Arousal, Valence, AU06, AU12). It is important to note that we generated another (control) sample with the same conditions (i.e., random 10% sampling balanced for obesity category) and we ran all models on this second subsample as well in order to investigate the representativeness of our reduced dataset. No differences were found in the results obtained between two subsamples (see Supplementary material). In addition, to support observation based analysis, we performed participant level control analysis. As a sensitivity analysis, the models were additionally fitted to participant × condition means. Standardized contrasts between obesity categories were calculated from these participant-level models by dividing estimated marginal mean contrasts by the model residual standard deviation, and are reported with 95% confidence intervals (for details see Supplementary material).

We analyzed the intensity of facial prosodic features by condition, obesity score and gender with linear mixed models [LMM, lme4 package, lme function, (36, 37)] with AIC-based backwards elimination [MASS package, drop1 function, (38)] to find parsimonious models. We analyzed the distribution of each variable with histograms and QQ plots. In line with the distributions, we applied LMM for Gaussian distribution with a log link function for all variables. After checking homogeneity of variance we controlled for variance of conditions in all models as dog-directed condition was significantly different from adult- and infant-directed condition (varIdent function). All models included condition (AD, DD, ID), obesity category (obescat: normal weight vs. overweight), gender (female, male) and their two-way interactions as fixed effects. Participant identity (new_ID) was included as a random intercept and condition was included as a participant-specific random slope. Thus, the random-effects structure allowed both baseline response levels and the effects condition to vary among participants. Heterogeneity of residual variance across conditions was accommodated using a VarIdent variance structure. Models were fitted using maximum likelihood estimation (for details see Supplementary material). For *post-hoc* pairwise comparisons, we used the Tukey method [emmeans package, (39)].

## Results

### Happy facial expression

The effect of obesity category differed across conditions, with a significant DD × obesity category interaction (b = −0.0601, SE = 0.0277, *t* = −2.17, *p* = 0.030), whereas the AD × obesity category interaction was not significant (*p* = 0.948). *Post-hoc* comparisons of estimated marginal means showed no difference between non-obese and obese participants in the ID condition (EMM = 0.320 vs. 0.326; estimated difference = −0.0064, SE = 0.0342, *t* = −0.19, *p* = 0.853) or in the AD condition (EMM = 0.335 vs. 0.339; estimated difference = −0.0041, SE = 0.0368, *t* = −0.11, *p* = 0.912). In contrast, in the DD condition, non-obese participants displayed higher Happy scores than obese participants (EMM = 0.230 vs. 0.176; estimated difference = 0.0538, SE = 0.0247, *t* = 2.18, *p* = 0.031; see Figure 1).

**Figure 1:**
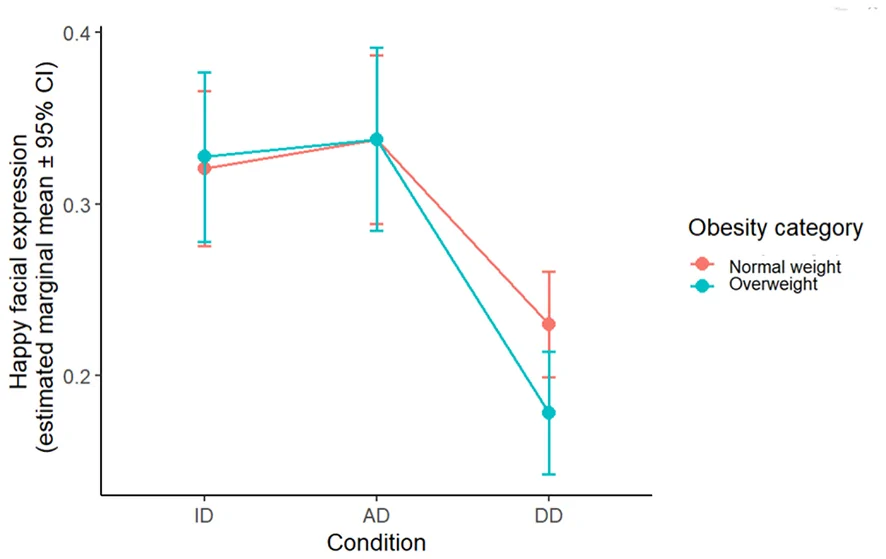
Intensity of happy facial expression when speaking to infants (ID), adults (AD), and dogs (DD) in normal weight (red) vs. overweight (blue) speakers.

Happy facial expression was lower in the DD condition than in the reference ID condition (b = −0.0902, SE = 0.0191, *t* = −4.72, *p* < 0.001), whereas the AD condition did not differ from ID (b = 0.0152, SE = 0.0241, *t* = 0.63, *p* = 0.528). Neither the main effect of obesity category (*p* = 0.779) nor gender (*p* = 0.093) was significant. The obesity category × gender interaction was not significant (b = −0.0085, SE = 0.0478, *t* = −0.18, *p* = 0.860).

### Facial arousal

Obesity category was significantly associated with Arousal in the fitted model, with obese participants showing lower Arousal scores than non-obese participants at the reference levels of the interacting factors (b = −0.0335, SE = 0.0148, *t* = −2.27, *p* = 0.025, see Figure 2). When estimated marginal means were averaged across communication conditions and gender, Arousal was also numerically lower in obese than in non-obese participants [EMM = 0.464, 95% CI [0.445, 0.482] vs. EMM = 0.480, 95% CI [0.464, 0.495]], although this averaged contrast was not significant (estimated difference = 0.0162, SE = 0.0121, t = 1.34, *p* = 0.182).

**Figure 2:**
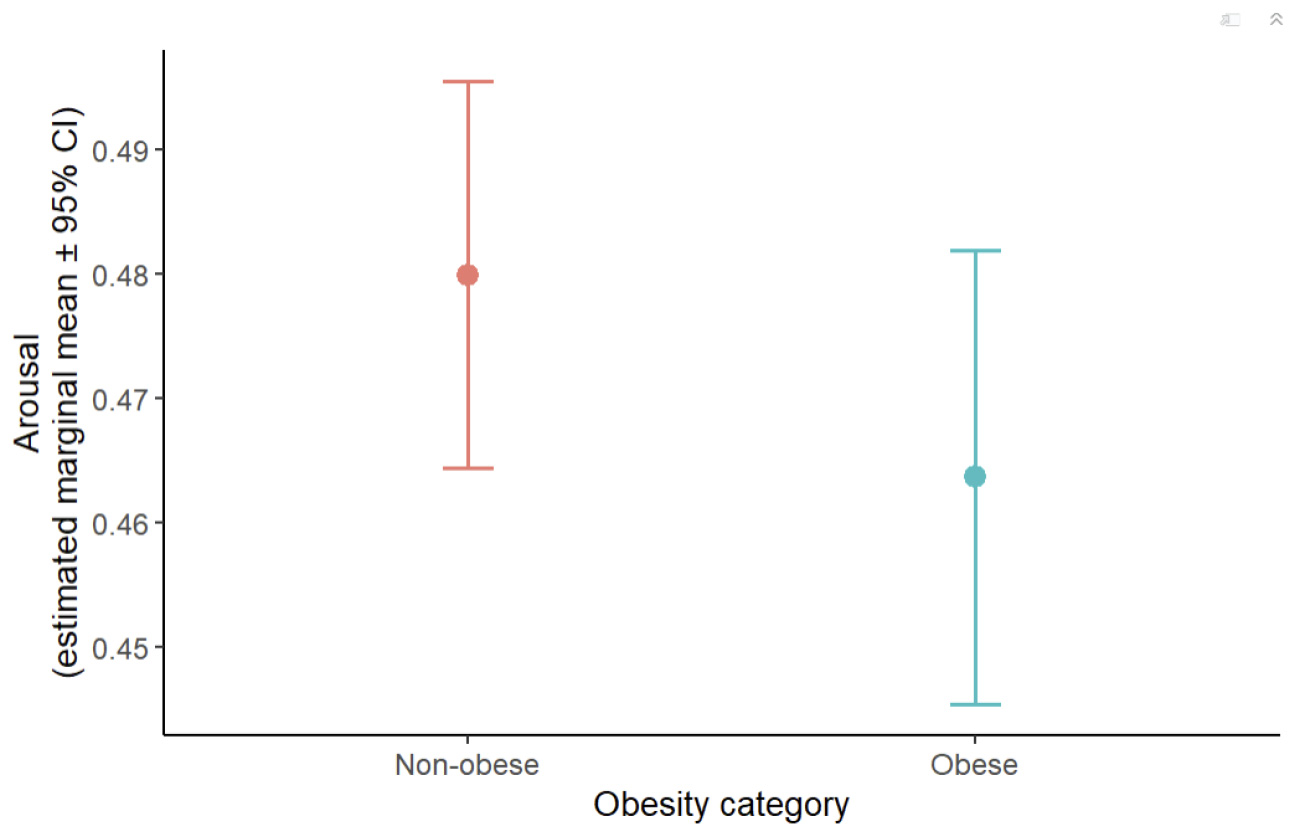
Intensity of facial arousal in normal (red) and overweight (blue) speakers.

Arousal also differed across communication conditions. Estimated marginal means were lower in the DD condition [EMM = 0.448, 95% CI [0.432, 0.463]] than in both the ID condition [EMM = 0.482, 95% CI [0.471, 0.494]; estimated difference = 0.0344, SE = 0.0063, *t* = 5.47, *p* < 0.001] and the AD condition [EMM = 0.485, 95% CI [0.470, 0.501]; estimated difference = 0.0377, SE = 0.0083, *t* = 4.53, *p* < 0.001], whereas ID and AD did not differ (*p* = 0.830).

Neither the condition × obesity category interaction (AD × obesity: b = 0.0091, SE = 0.0114, *t* = 0.80, *p* = 0.422; DD × obesity: b = −0.0121, SE = 0.0126, *t* = −0.96, *p* = 0.337] nor the obesity category × gender interaction (b = 0.0365, SE = 0.0230, *t* = 1.59, *p* = 0.115) was significant. Gender did not reach statistical significance (b = −0.0292, SE = 0.0151, *t* = −1.94, *p* = 0.055).

### Valence of facial expressions

Similarly to Happy facial expression, Valence differed according to obesity category specifically in the DD condition, as indicated by a significant DD × obesity category interaction (b = −0.0782, SE = 0.0375, *t* = −2.09, *p* = 0.037). *Post-hoc* comparisons showed no difference between non-obese and obese participants in the ID condition (EMM = 0.171 vs. 0.173; estimated difference = −0.0014, SE = 0.0443, *t* = −0.03, *p* = 0.976) or in the AD condition (EMM = 0.229 vs. 0.232; estimated difference = −0.0030, SE = 0.0450, *t* = −0.07, *p* = 0.947). In contrast, in the DD condition, non-obese participants showed higher Valence scores than obese participants (EMM = 0.061 vs. −0.016; estimated difference = 0.0768, SE = 0.0339, *t* = 2.26, *p* = 0.025, see Figure 3).

**Figure 3:**
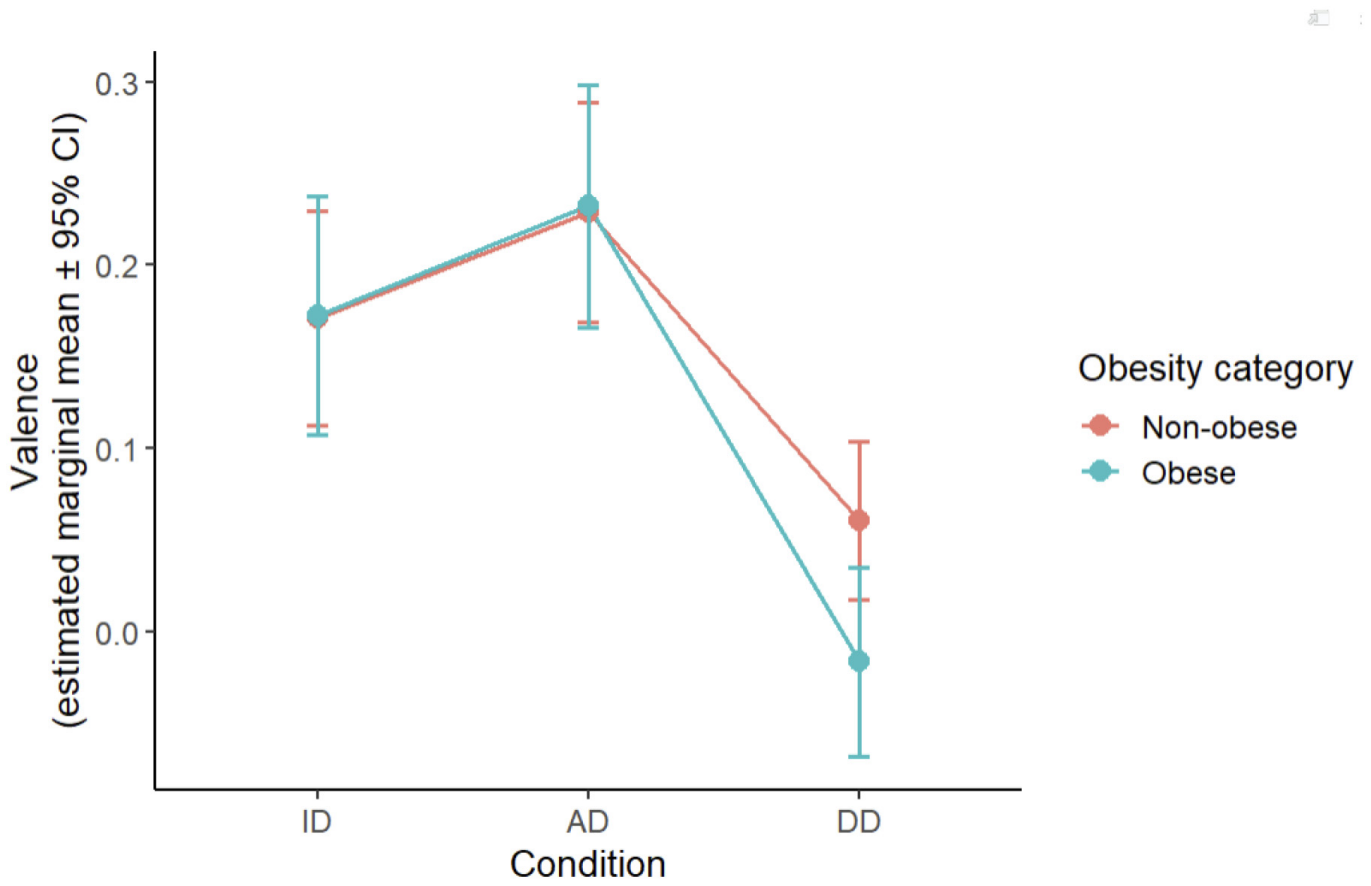
Valence of facial expression when speaking to infants (ID), adults (AD), and dogs (DD) in normal (red) and overweight (blue) speakers.

Gender was also associated with Valence, with female participants showing higher estimated marginal Valence than male participants [EMM = 0.198, 95% CI [0.159, 0.237] vs. EMM = 0.085, 95% CI [0.035, 0.135]; estimated difference = 0.113, SE = 0.0311, *t* = 3.63, *p* < 0.001]. The obesity category × gender interaction was not significant (b = −0.0327, SE = 0.0622, *t* = −0.53, *p* = 0.599).

### Intensity of cheek raiser action unit (AU6)

AU6 differed across communication conditions. Estimated marginal means were lower in the DD condition [EMM = 0.197, 95% CI [0.180, 0.214]] than in both the ID condition [EMM = 0.253, 95% CI [0.229, 0.277]; estimated difference = 0.0554, SE = 0.0115, *t* = 4.80, *p* < 0.001] and the AD condition [EMM = 0.263, 95% CI [0.236, 0.290]; estimated difference = 0.0658, SE = 0.0118, *t* = 5.57, *p* < 0.001]. The ID and AD conditions did not differ (*p* = 0.732, see Figure 4).

**Figure 4:**
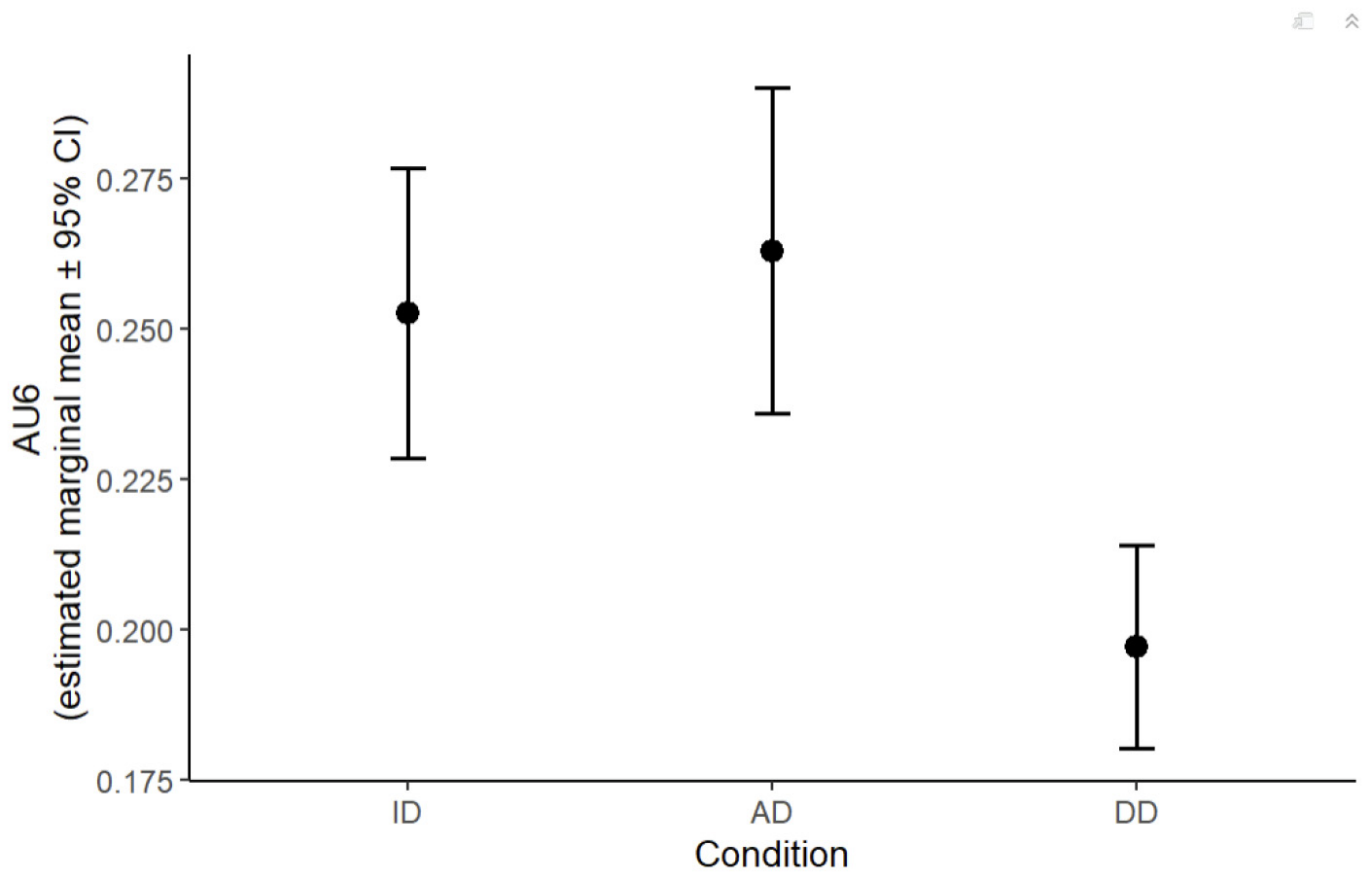
Intensity of cheek raiser muscle across infant (ID), adult (AD), and dog (DD) directed communication.

Neither obesity category (b = 0.0319, SE = 0.0272, *t* = 1.17, *p* = 0.243) nor gender (b = 0.0045, SE = 0.0209, *t* = 0.21, *p* = 0.830) showed a significant main effect. Neither the condition × obesity category interaction (AD × obesity: *p* = 0.219; DD × obesity: *p* = 0.112) nor the obesity category × gender interaction (*p* = 0.724) was significant.

### Effect size

Standardized effect sizes at the participant level were calculated for the obesity-category contrasts within each communication condition and are reported with 95% confidence intervals in Table 2. The participant-level standardized estimates indicated that obesity-category differences varied across communication conditions and outcome measures, with the largest standardized difference observed for Arousal in the DD condition [0.798, 95% CI [0.282, 1.315]].

**Table 2:** Values are standardized estimated marginal mean contrasts between obesity categories (category 1–category 2), with 95% confidence intervals.

Outcome	ID	AD	DD
Happy	−0.068 [−0.628, 0.491]	−0.006 [−0.562, 0.551]	0.447 [−0.036, 0.930]
Arousal	0.380 [−0.211, 0.971]	0.357 [−0.231, 0.945]	**0.798 [0.282, 1.315]**
Valence	−0.064 [−0.609, 0.482]	−0.076 [−0.619, 0.467]	0.465 [−0.004, 0.934]
AU6	−0.204 [−0.738, 0.329]	**−0.547 [−1.077**, **−0.016]**	0.084 [−0.372, 0.540]

Significant results are highlighted in bold.

## Discussion

The present study investigated whether body condition is associated with variation in visual prosody during dog-directed communication. Consistent with our predictions, body condition scores co-varied with differences in facial prosodic displays. However, these effects were not uniform across facial variables or communication contexts. Instead, the influence of body condition depended on the interaction partner, suggesting that body shape-related variation in visible facial prosody manifests differently during dog-, infant-, and adult-directed communication. Most notably, differences in positive facial expressions emerged specifically during dog-directed interactions, whereas reduced facial arousal was observed across all communication partners.

Our findings only partially support the hypothesis that speakers with higher body condition scores would display generally less intense facial prosodic signals. The clearest support for this prediction was observed in facial arousal, which was consistently lower among speakers with higher body condition scores regardless of whether they interacted with a dog, infant, or adult. This result aligns with previous literature suggesting altered emotional processing and expression in overweight and obese individuals (20, 22, 23). One possible explanation for this finding is the anatomical constraint of excess facial adipose tissue masking the salience of facial muscle movements. Alternatively or in parallel, reduced facial arousal may reflect a lower tendency to externally express emotional states, a greater degree of expressive suppression, or differences in emotion regulation strategies. Similar patterns have been reported in studies demonstrating attenuated facial reactions to emotionally salient stimuli among overweight and obese individuals (24). Our results extend these findings by showing that such differences are detectable not only during controlled laboratory tasks but also during naturalistic social interactions.

Interestingly, body shape-related differences in positive emotional displays were not equally present across communication partners. Happy facial expression and facial valence differed between body condition groups only during dog-directed communication, whereas no such differences emerged during infant- or adult-directed interactions. This suggests that dog-directed communication may be particularly sensitive to obesity-related variation in visual prosody. This finding was unexpected because previous studies have generally highlighted similarities between dog-directed and infant-directed communication (2, 4). The present results suggest that, despite these similarities, visual communication toward dogs may be regulated differently than communication toward human partners.

One possible explanation is that body shape-related differences become particularly apparent in dog-directed communication because visual signaling toward dogs differs fundamentally from signaling toward humans. Previous studies have demonstrated that people generally display less intense positive facial expressions and reduced activation of several facial muscles when communicating with dogs compared to infants or adults (2, 4). We previously suggested that this pattern may reflect an evolutionary mismatch between human and canine visual communication systems. Several facial cues that humans typically associate with positive emotions, such as visible teeth and increased visibility of the sclera, can serve agonistic or threatening functions in canids and wolves. Consequently, humans may attenuate certain facial signals when interacting with dogs in order to maintain communicative clarity and avoid transmitting potentially ambiguous information. From this perspective, dog-directed communication may already operate within a narrower expressive range than human-directed communication. Additional factors affecting facial expression, such as obesity-related differences in emotional expression or facial morphology, may therefore become more apparent in this context. This interpretation could explain why body shape-related differences in happy facial expression and Valence emerged specifically during dog-directed communication, despite the substantial similarities previously reported between dog- and infant-directed prosody. Rather than representing a general reduction in positive emotional expression, general body condition may interact with species-specific communicative constraints that characterize human-dog interactions. An alternative, non-exclusive explanation is that dogs rely heavily on facial and emotional signals during social interactions (16), potentially making subtle differences in visual prosody more relevant in dog-directed communication than in communication with human partners. Future studies investigating how dogs respond behaviorally to body shape-related variation in owner facial expressions could help disentangle these possibilities.

The results concerning AU6 (cheek raiser) intensity further highlight the complexity of the relationship between body condition and visual prosody. Contrary to our initial prediction, speakers with higher body shape scores displayed stronger cheek raiser action unit when communicating with infants and adults, whereas no difference was found during dog-directed interactions. If body condition merely reduced the visibility of facial movements, lower rather than higher AU6 intensity would be expected. Instead, the observed pattern suggests that obesity influences different components of facial expression in distinct ways.

There are several different explanations we would like to consider for these findings. Firstly, facial morphology may have altered the mapping between facial movement and FaceReader output. That is, muscles might have been activated without producing a visible skin displacement due to the intervening soft tissue under the skin. Secondly, the differences in FaceReader measurements might have to do with model quality. The program might have modeled faces with different shapes and sizes differently, leading to differences in the final output. Thirdly, these results might reflect differences in facial expressivity, as opposed to differences in emotional processing. That is, subject groups might have only differed in their habitual communicative style. Fourthly, the communication contexts may have amplified or masked body-condition differences. Finally, there might be a compensation mechanism in individuals with higher overall body-condition scores due to the reduced muscle activity or emotional expressivity that manifests as increased AU06 intensity. It is important to note, however, that no direct muscle activation was measured here.

Further research using more detailed facial action analyses, as well as direct measures of muscle activation, such as EMG, will be needed to clarify the functional significance of this pattern.

The observed gender differences replicated and extended previous findings on visual prosody. Female speakers displayed more intense positive facial expressions and greater facial arousal than male speakers in several communication contexts. Earlier studies reported similar tendencies but often lacked sufficient statistical power to detect robust gender effects (2, 4). By combining data from multiple datasets, the present study was able to reveal more consistent gender-related variation.

More broadly, the present findings contribute to the growing literature emphasizing the importance of visual signals in dog-human communication. Dogs form attachment relationships with their owners that resemble human infant-caregiver bonds in several respects (7, 8), and many owners interact with their dogs using communication styles similar to those directed toward young children (9). Previous research has demonstrated that facial prosody is a characteristic feature of both dog-directed and infant-directed communication (2, 4) (Koós-Hutás et al., in prep). Our results suggest that individual characteristics of the speaker, including general body condition and gender, also contribute to variation in these signals. Consequently, dog-directed communication should not be viewed as a fixed behavioral pattern but rather as a flexible communicative system shaped by both situational and individual factors.

Several limitations should be considered when interpreting the present findings. First, the present study is inherently correlational and therefore does not permit causal conclusions regarding the mechanisms underlying the observed differences. Second, body shape was assessed using visual body shape categories rather than directly measured body composition. Although the categories were based on established clinical guidelines, more precise anthropometric measures would strengthen future studies. Third, due to the random selection of participants for the original studies comprising the present dataset, the body shape groups were unevenly represented. Fourth, computational limitations required the analysis of a balanced random subsample of the full dataset. Although analyses on an independently generated control subsample yielded identical conclusions, future studies using the complete dataset would provide additional confirmation. In the present study missing values were excluded from our data (i.e., those moments in which FaceReader was not able to detect facial movements of the speaker). It is important to note, however, that body composition can be one possible cause of non-detectable facial movements, therefore a difference in the ratio of missing values could emerge between obese and non-obese participants. To investigate this possibility is out of the scope of the present paper, yet it can be an interesting aim for upcoming studies on facial prosody and body condition in relation to automatic facial coding software development.

In summary, general body condition was associated with variation in visual prosody, but these effects depended strongly on the communication partner and the facial variable under investigation. While higher body condition scores were consistently associated with lower facial arousal, differences in positive emotional displays emerged primarily during dog-directed communication. These findings suggest that body shape-related variation in emotional expression extends to human-animal interactions and highlight the importance of considering individual characteristics of the speaker when investigating dog-directed communication. More generally, the results support the view that visual prosody represents an important yet understudied component of dog-human communication and may contribute to the quality of interactions between dogs and their owners.

## Data Availability

The raw data supporting the conclusions of this article will be made available by the authors, without undue reservation.
