# Supplementary material for: Body condition-linked alterations in dog owners' visual prosody
Source: Front Vet Sci. 2026 Oct 28;13:1962197. doi: 10.3389/fvets.2026.1962197 (PMC13752995; doi:10.3389/fvets.2026.1962197)
Supplement: Supplementary file 1 [file Table_1.docx]

Supplementary Material
Mixed-effects model results

For each outcome, the fixed-effects structure included communication condition, obesity category, and gender as main effects, together with condition × obesity category and obesity category × gender interactions. Models were fitted using maximum likelihood. Participant-specific random slopes for condition were compared with otherwise identical random-intercept-only models using likelihood-ratio tests.

# Happy

## Table S1. Fixed effects from the final Happy model

| **Effect** | **Estimate** | **SE** | **df** | **t** | **p** |
| --- | --- | --- | --- | --- | --- |
| (Intercept) | 0.345714 | 0.026218 | 95593 | 13.186 | < .001 |
| conditionAD | 0.015194 | 0.024097 | 95593 | 0.631 | .528 |
| conditionDD | -0.090245 | 0.019123 | 95593 | -4.719 | < .001 |
| obescat2 | 0.010579 | 0.037550 | 140 | 0.282 | .779 |
| gender2 | -0.051347 | 0.030379 | 140 | -1.690 | .093 |
| conditionAD:obescat2 | -0.002280 | 0.035069 | 95593 | -0.065 | .948 |
| conditionDD:obescat2 | -0.060110 | 0.027712 | 95593 | -2.169 | .030 |
| obescat2:gender2 | -0.008457 | 0.047758 | 140 | -0.177 | .860 |

## Table S2. Random-effects structure comparison

| **Model** | **df** | **AIC** | **BIC** | **logLik** | **LRT χ²** | **p** |
| --- | --- | --- | --- | --- | --- | --- |
| Intercept only | 12 | 16258.99 | 16372.63 | -8117.497 |  |  |
| Condition random slope | 17 | 6878.73 | 7039.71 | -3422.365 | 9390.263 | < .0001 |

*Likelihood-ratio test: χ²(5) = 9390.263, p < .0001.*

# Arousal

## Table S3. Fixed effects from the final Arousal model

| **Effect** | **Estimate** | **SE** | **df** | **t** | **p** |
| --- | --- | --- | --- | --- | --- |
| (Intercept) | 0.504398 | 0.010832 | 95593 | 46.566 | < .001 |
| conditionAD | -0.001255 | 0.007790 | 95593 | -0.161 | .872 |
| conditionDD | -0.028362 | 0.008609 | 95593 | -3.295 | .001 |
| obescat2 | -0.033495 | 0.014769 | 140 | -2.268 | .025 |
| gender2 | -0.029215 | 0.015087 | 140 | -1.936 | .055 |
| conditionAD:obescat2 | 0.009116 | 0.011358 | 95593 | 0.803 | .422 |
| conditionDD:obescat2 | -0.012081 | 0.012581 | 95593 | -0.960 | .337 |
| obescat2:gender2 | 0.036480 | 0.022974 | 140 | 1.588 | .115 |

## Table S4. Random-effects structure comparison

| **Model** | **df** | **AIC** | **BIC** | **logLik** | **LRT χ²** | **p** |
| --- | --- | --- | --- | --- | --- | --- |
| Intercept only | 12 | -93571.54 | -93457.91 | 46797.77 |  |  |
| Condition random slope (diagonal) | 14 | -98428.63 | -98296.06 | 49228.31 | 4861.089 | < .0001 |

*Likelihood-ratio test: χ²(2) = 4861.089, p < .0001.*

# Valence

## Table S5. Fixed effects from the final Valence model

| **Effect** | **Estimate** | **SE** | **df** | **t** | **p** |
| --- | --- | --- | --- | --- | --- |
| (Intercept) | 0.219517 | 0.034506 | 95593 | 6.362 | < .001 |
| conditionAD | 0.057908 | 0.030826 | 95593 | 1.879 | .060 |
| conditionDD | -0.110462 | 0.025843 | 95593 | -4.274 | < .001 |
| obescat2 | 0.017728 | 0.049136 | 140 | 0.361 | .719 |
| gender2 | -0.096638 | 0.039883 | 140 | -2.423 | .017 |
| conditionAD:obescat2 | 0.001649 | 0.044870 | 95593 | 0.037 | .971 |
| conditionDD:obescat2 | -0.078154 | 0.037460 | 95593 | -2.086 | .037 |
| obescat2:gender2 | -0.032741 | 0.062201 | 140 | -0.526 | .599 |

## Table S6. Random-effects structure comparison

| **Model** | **df** | **AIC** | **BIC** | **logLik** | **LRT χ²** | **p** |
| --- | --- | --- | --- | --- | --- | --- |
| Intercept only | 12 | 65794.99 | 65908.62 | -32885.49 |  |  |
| Condition random slope | 17 | 55721.21 | 55882.19 | -27843.60 | 10083.78 | < .0001 |

*Likelihood-ratio test: χ²(5) = 10083.78, p < .0001.*

# AU6

## Table S7. Fixed effects from the final AU6 model

| **Effect** | **Estimate** | **SE** | **df** | **t** | **p** |
| --- | --- | --- | --- | --- | --- |
| (Intercept) | 0.237335 | 0.019234 | 95593 | 12.339 | < .001 |
| conditionAD | -0.006564 | 0.018982 | 95593 | -0.346 | .730 |
| conditionDD | -0.037086 | 0.015908 | 95593 | -2.331 | .020 |
| obescat2 | 0.031895 | 0.027209 | 140 | 1.172 | .243 |
| gender2 | 0.004483 | 0.020883 | 140 | 0.215 | .830 |
| conditionAD:obescat2 | 0.033934 | 0.027613 | 95593 | 1.229 | .219 |
| conditionDD:obescat2 | -0.036623 | 0.023073 | 95593 | -1.587 | .112 |
| obescat2:gender2 | -0.011613 | 0.032768 | 140 | -0.354 | .724 |

## Table S8. Random-effects structure comparison

| **Model** | **df** | **AIC** | **BIC** | **logLik** | **LRT χ²** | **p** |
| --- | --- | --- | --- | --- | --- | --- |
| Intercept only | 12 | -28115.76 | -28002.12 | 14069.88 |  |  |
| Condition random slope | 17 | -37269.95 | -37108.97 | 18651.97 | 9164.191 | < .0001 |

*Likelihood-ratio test: χ²(5) = 9164.191, p < .0001.*

**Note.** For Arousal, the fully correlated condition random-slope covariance structure could not be estimated reliably because of a singular precision matrix. The final model therefore used a diagonal random-effects covariance structure [pdDiag(~ condition)], allowing participant-specific condition-slope variances without estimating correlations among the random effects.

# Participant-level standardized effect sizes

Standardized effect sizes at the participant level were calculated for the obesity-category contrasts within each communication condition and are reported with 95% confidence intervals in Table S9. The participant-level standardized estimates indicated that obesity-category differences varied across communication conditions and outcome measures, with the largest standardized difference observed for Arousal in the DD condition (0.798, 95% CI [0.282, 1.315]).

**Table S9. Participant-level standardized effect sizes for obesity-category contrasts**

| **Outcome** | **ID** | **AD** | **DD** |
| --- | --- | --- | --- |
| Happy | −0.068 [−0.628, 0.491] | −0.006 [−0.562, 0.551] | 0.447 [−0.036, 0.930] |
| Arousal | 0.380 [−0.211, 0.971] | 0.357 [−0.231, 0.945] | **0.798 [0.282, 1.315]** |
| Valence | −0.064 [−0.609, 0.482] | −0.076 [−0.619, 0.467] | 0.465 [−0.004, 0.934] |
| AU6 | −0.204 [−0.738, 0.329] | **−0.547 [−1.077, −0.016]** | 0.084 [−0.372, 0.540] |

**Note.** Values are standardized estimated marginal mean contrasts between obesity categories (category 1 − category 2), with 95% confidence intervals.
