# Supplementary material for: Body condition-linked alterations in dog owners' visual prosody
Source: Front Vet Sci. 2026 Oct 28;13:1962197. doi: 10.3389/fvets.2026.1962197 (PMC13752995; doi:10.3389/fvets.2026.1962197)
Supplement: Supplementary file 2 [file Table_2.docx]

---

title: "R Notebook"

output: html_notebook

---

```{r}

```

```{r}

library(tidyverse)

library(readxl)

library(performance)

library(easystats)

library(interactions)

library(jtools)

library(emmeans)

library(ggeffects)

library(ggstatsplot)

library(lme4)

library(lmerTest)

library(nlme)

library(sjPlot)

library(car)

library(dplyr)

library(ggplot2)

```

```{r}

obespros <- read_excel("obes_prosody_alldata_nofitfailed.xlsx", na = c("FIT_FAILED", "FIND_FAILED")) %>%

mutate(across(where(is_character),as_factor))

#inspect data

#[str. should be 964610 obs. of 46 var.]

str(obespros)

head(obespros)

tail(obespros)

#number of missing values [should be 1880113]

sum(is.na(obespros))

#transform variables from num to factor

obespros$gender <- as.factor(obespros$gender)

obespros$obescat <- as.factor(obespros$obescat)

obespros$study <- as.factor(obespros$study)

#sample 5000 random observations for easier debugging

s_data <- filter(obespros) %>% sample_n(5000)

#sample of 10% random observations from each obesity score

s_data_obscore <- filter(obespros) %>%

group_by(obesscore) %>%

sample_frac(0.1)

#checking if reduced data is balanced

library(dplyr)

n_distinct(obespros$new_ID)

n_distinct(s_data_obscore$new_ID)

setdiff(

unique(obespros$new_ID),

unique(s_data_obscore$new_ID)

)

```

```{r}

\# histograms

hist(s_data_obscore$Happy)

hist(s_data_obscore$Neutral)

hist(s_data_obscore$Arousal)

hist(s_data_obscore$Valence)

hist(s_data$Happy)

hist(s_data$Neutral)

hist(s_data$Arousal)

hist(s_data$Valence)

```

\#### random slope models

###HAppy

```{r}

random = ~ condition | new_ID

#HAppy

\# Random-intercept-only model

model_happy_intercept <- lme(

Happy ~ condition + obescat + gender +

condition:obescat +

obescat:gender,

random = ~ 1 | new_ID,

data = s_data_obscore,

method = "ML",

weights = varIdent(form = ~1 | condition),

na.action = na.exclude

)

\# Compare random-intercept and random-slope models

anova(model_happy_intercept, model_happy_final)

#final model

model_happy_final <- lme(

Happy ~ condition + obescat + gender +

condition:obescat +

obescat:gender,

random = ~ condition | new_ID,

data = s_data_obscore,

method = "ML",

weights = varIdent(form = ~1 | condition),

na.action = na.exclude,

control = lmeControl(

opt = "optim",

maxIter = 100,

msMaxIter = 100

)

)

summary(model_happy_final)$tTable

emm_happy_obes <- emmeans(

model_happy_final,

~ obescat | condition

)

emm_happy_obes

pairs(emm_happy_obes)

\# Plot

ggplot(

happy_obes_plot,

aes(

x = condition,

y = emmean,

group = obescat,

colour = obescat

)

) +

geom_line(

linewidth = 0.8

) +

geom_point(

size = 3.5

) +

geom_errorbar(

aes(

ymin = lower.CL,

ymax = upper.CL

),

width = 0.08,

linewidth = 0.8

) +

scale_colour_manual(

values = c(

"Non-obese" = "#F8766D",

"Obese" = "#00BFC4"

)

) +

labs(

x = "Condition",

y = "Happy facial expression\n (estimated marginal mean ± 95% CI)",

colour = "Obesity category"

) +

theme_classic(base_size = 14) +

theme(

legend.title = element_text(size = 14),

legend.text = element_text(size = 12),

axis.title = element_text(size = 14),

axis.text = element_text(size = 12)

)

```

###Arousal

```{r}

#Arousal

\# Random-intercept-only model

model_arousal_intercept <- lme(

Arousal ~ condition + obescat + gender +

condition:obescat +

obescat:gender,

random = ~ 1 | new_ID,

data = s_data_obscore,

method = "ML",

weights = varIdent(form = ~1 | condition),

na.action = na.exclude

)

\# Compare random-intercept and random-slope models

anova(model_arousal_intercept, model_arousal_final)

#final model

model_arousal_final <- lme(

Arousal ~ condition + obescat + gender +

condition:obescat +

obescat:gender,

random = list(

new_ID = pdDiag(~ condition)

),

data = s_data_obscore,

method = "ML",

weights = varIdent(form = ~1 | condition),

na.action = na.exclude,

control = lmeControl(

opt = "optim",

maxIter = 200,

msMaxIter = 200

)

)

summary(model_arousal_final)$tTable

emm_arousal_obes <- emmeans(

model_arousal_final,

~ obescat

)

emm_arousal_obes

pairs(emm_arousal_obes)

emm_arousal_condition <- emmeans(

model_arousal_final,

~ condition

)

emm_arousal_condition

pairs(emm_arousal_condition)

#plot

arousal_obes_plot <- as.data.frame(emm_arousal_obes)

arousal_obes_plot$obescat <- factor(

arousal_obes_plot$obescat,

levels = c("1", "2"),

labels = c("Non-obese", "Obese")

)

windows(width = 8, height = 7)

ggplot(

arousal_obes_plot,

aes(x = obescat, y = emmean, colour = obescat)

) +

geom_point(size = 4) +

geom_errorbar(

aes(ymin = lower.CL, ymax = upper.CL),

width = 0.10,

linewidth = 0.8

) +

scale_colour_manual(

values = c(

"Non-obese" = "#F8766D",

"Obese" = "#00BFC4"

)

) +

labs(

x = "Obesity category",

y = "Arousal\n(estimated marginal mean ± 95% CI)",

colour = "Obesity category"

) +

theme_classic(base_size = 14) +

theme(

legend.position = "none",

axis.title = element_text(size = 14),

axis.text = element_text(size = 12)

)

```

###Valence

```{r}

\# slope vs intercept model

model_valence_intercept <- lme(

Valence ~ condition + obescat + gender +

condition:obescat +

obescat:gender,

random = ~ 1 | new_ID,

data = s_data_obscore,

method = "ML",

weights = varIdent(form = ~1 | condition),

na.action = na.exclude

)

anova(model_valence_intercept, model_valence_final)

\# final model

model_valence_final <- lme(

Valence ~ condition + obescat + gender +

condition:obescat +

obescat:gender,

random = ~ condition | new_ID,

data = s_data_obscore,

method = "ML",

weights = varIdent(form = ~1 | condition),

na.action = na.exclude,

control = lmeControl(

opt = "optim",

maxIter = 200,

msMaxIter = 200

)

)

summary(model_valence_final)$tTable

\# Obesity within each condition

emm_valence_obes <- emmeans(

model_valence_final,

~ obescat | condition

)

emm_valence_obes

pairs(emm_valence_obes)

\# Gender

emm_valence_gender <- emmeans(

model_valence_final,

~ gender

)

emm_valence_gender

pairs(emm_valence_gender)

#plot

valence_obes_plot <- as.data.frame(emm_valence_obes)

valence_obes_plot$obescat <- factor(

valence_obes_plot$obescat,

levels = c("1", "2"),

labels = c("Non-obese", "Obese")

)

windows(width = 10, height = 7)

ggplot(

valence_obes_plot,

aes(

x = condition,

y = emmean,

group = obescat,

colour = obescat

)

) +

geom_line(linewidth = 0.8) +

geom_point(size = 3.5) +

geom_errorbar(

aes(ymin = lower.CL, ymax = upper.CL),

width = 0.08,

linewidth = 0.8

) +

scale_colour_manual(

values = c(

"Non-obese" = "#F8766D",

"Obese" = "#00BFC4"

)

) +

labs(

x = "Condition",

y = "Valence\n(estimated marginal mean ± 95% CI)",

colour = "Obesity category"

) +

theme_classic(base_size = 14) +

theme(

legend.title = element_text(size = 14),

legend.text = element_text(size = 12),

axis.title = element_text(size = 14),

axis.text = element_text(size = 12)

)

```

###Action Unit 6

```{r}

#slope vs intercept model

model_AU6_intercept <- lme(

AU6 ~ condition + obescat + gender +

condition:obescat +

obescat:gender,

random = ~ 1 | new_ID,

data = s_data_obscore,

method = "ML",

weights = varIdent(form = ~1 | condition),

na.action = na.exclude

)

anova(model_AU6_intercept, model_AU6_final)

\# final model

model_AU6_final <- lme(

AU6 ~ condition + obescat + gender +

condition:obescat +

obescat:gender,

random = ~ condition | new_ID,

data = s_data_obscore,

method = "ML",

weights = varIdent(form = ~1 | condition),

na.action = na.exclude,

control = lmeControl(

opt = "optim",

maxIter = 200,

msMaxIter = 200

)

)

summary(model_AU6_final)$tTable

emm_AU6_condition <- emmeans(

model_AU6_final,

~ condition

)

emm_AU6_condition

pairs(emm_AU6_condition, adjust = "tukey")

#plot

AU6_condition_plot <- as.data.frame(emm_AU6_condition)

windows(width = 8, height = 7)

ggplot(

AU6_condition_plot,

aes(x = condition, y = emmean)

) +

geom_point(size = 4) +

geom_errorbar(

aes(ymin = lower.CL, ymax = upper.CL),

width = 0.10,

linewidth = 0.8

) +

labs(

x = "Condition",

y = "AU6\n(estimated marginal mean ± 95% CI)"

) +

theme_classic(base_size = 14) +

theme(

axis.title = element_text(size = 14),

axis.text = element_text(size = 12)

)

```

\### participant level vs observation (frame) level analysis

```{r}

participant_condition <- s_data_obscore %>%

group_by(new_ID, condition, obescat, gender) %>%

summarise(

Happy = mean(Happy, na.rm = TRUE),

.groups = "drop"

)

dim(participant_condition)

table(participant_condition$condition)

n_distinct(participant_condition$new_ID)

random = ~1 | new_ID

model2_participant <- lme(

Happy ~ condition + obescat + gender +

condition:obescat + condition:gender,

random = ~1 | new_ID,

data = participant_condition,

method = "ML",

na.action = na.exclude

)

summary (model2_participant)

summary(model2_participant)$tTable

VarCorr(model2_participant)

```

\### effect size

```{r}

library(emmeans)

library(effectsize)

emm_part <- emmeans(

model2_participant,

~ obescat | condition

)

emm_part

pairs(emm_part)

eff_part <- eff_size(

emm_part,

sigma = sigma(model2_participant),

edf = Inf

)

eff_part

library(dplyr)

library(nlme)

library(emmeans)

outcomes <- c("Happy", "Arousal", "Valence", "AU6")

results_effectsize <- list()

results_models <- list()

for (outcome in outcomes) {

\# Participant × condition averages

dat_pc <- s_data_obscore %>%

group_by(new_ID, condition, obescat, gender) %>%

summarise(

outcome_mean = mean(.data[[outcome]], na.rm = TRUE),

.groups = "drop"

) %>%

filter(is.finite(outcome_mean))

\# Participant × condition model

model_pc <- lme(

outcome_mean ~ condition + obescat + gender +

condition:obescat + condition:gender,

random = ~1 | new_ID,

data = dat_pc,

method = "ML",

na.action = na.exclude

)

\# Save model

results_models[[outcome]] <- model_pc

\# Estimated marginal means: obesity categories within condition

emm <- emmeans(

model_pc,

~ obescat | condition

)

\# Standardized contrasts + 95% CI

eff <- eff_size(

emm,

sigma = sigma(model_pc),

edf = Inf

)

\# Convert to data frame and add outcome name

eff_df <- as.data.frame(eff)

eff_df$outcome <- outcome

results_effectsize[[outcome]] <- eff_df

}

\# Combine all outcomes into one table

effectsize_table <- bind_rows(results_effectsize)

effectsize_table

effectsize_final <- bind_rows(

lapply(names(results_effectsize), function(x) {

tmp <- as.data.frame(results_effectsize[[x]])

data.frame(

Outcome = x,

Condition = tmp$condition,

Contrast = tmp$contrast,

Effect_size = tmp$effect.size,

SE = tmp$SE,

df = tmp$df,

CI_lower = tmp$lower.CL,

CI_upper = tmp$upper.CL

)

})

)

effectsize_final

```

\#### follow up analysis on another subsample in order to control for representativeness of s_data_obscore dataset

```{r}

s_data_obscore2 <- filter(obespros) %>%

group_by(obesscore) %>%

sample_frac(0.1)

model0 <- lme(Valence ~ condition + obescat + gender + condition:obescat, random = ~1|new_ID, data=s_data_obscore2, method = "ML", weights=varIdent(form = ~ 1|condition), na.action=na.exclude)

summary(model0)

tab_model(model0)

emmeans(model0, pairwise ~ obescat|condition)

emmeans(model0, pairwise ~ condition|obescat)

```

\#### residual temporal autocorrelation

```{r}

names(s_data_obscore)

head(s_data_obscore$`Video Time`, 20)

class(s_data_obscore$`Video Time`)

library(lubridate)

library(nlme)

s_data_obscore$video_sec <- period_to_seconds(

hms(as.character(s_data_obscore$`Video Time`))

)

head(s_data_obscore[, c("Video Time", "video_sec")])

s_data_obscore$recording_ID <- interaction(

s_data_obscore$new_ID,

s_data_obscore$condition,

drop = TRUE

)

model2_CAR1 <- lme(

Happy ~ condition + obescat + gender +

condition:obescat + condition:gender,

random = ~1 | new_ID,

correlation = corCAR1(

form = ~ video_sec | new_ID/condition

),

data = s_data_obscore,

method = "ML",

weights = varIdent(form = ~1 | condition),

na.action = na.exclude

)

library(dplyr)

s_data_obscore %>%

count(new_ID, condition, video_sec) %>%

filter(n > 1) %>%

arrange(desc(n)) %>%

head(20)

s_data_obscore %>%

count(new_ID, condition, video_sec) %>%

filter(n > 1) %>%

summarise(

duplicated_times = n(),

extra_rows = sum(n - 1)

)

s_data_obscore %>%

filter(duplicated(paste(new_ID, condition, video_sec)) |

duplicated(paste(new_ID, condition, video_sec), fromLast = TRUE)) %>%

select(new_ID, condition, `Video Time`, video_sec, obesscore) %>%

arrange(new_ID, condition, video_sec) %>%

head(20)

s_CAR1 <- s_data_obscore %>%

group_by(new_ID, condition, video_sec, obescat, gender) %>%

summarise(

Happy = mean(Happy, na.rm = TRUE),

.groups = "drop"

)

s_CAR1 %>%

count(new_ID, condition, video_sec) %>%

filter(n > 1)

model2_CAR1 <- lme(

Happy ~ condition + obescat + gender +

condition:obescat + condition:gender,

random = ~1 | new_ID,

correlation = corCAR1(

form = ~ video_sec | new_ID/condition

),

data = s_CAR1,

method = "ML",

weights = varIdent(form = ~1 | condition),

na.action = na.exclude

)

spacing <- s_CAR1 %>%

arrange(new_ID, condition, video_sec) %>%

group_by(new_ID, condition) %>%

mutate(time_gap = video_sec - lag(video_sec)) %>%

ungroup() %>%

filter(!is.na(time_gap))

summary(spacing$time_gap)

mean(spacing$time_gap <= 0.04) * 100

quantile(spacing$time_gap,

probs = c(.25, .5, .75, .9, .95))

```
